# Moderating the impact of patent linkage on access to medicines: lessons from variations in South Korea, Australia, Canada, and the United States

**DOI:** 10.1186/s12992-018-0423-0

**Published:** 2018-10-24

**Authors:** Kyung-Bok Son, Ruth Lopert, Deborah Gleeson, Tae-Jin Lee

**Affiliations:** 1 College of Pharmacy, Ewha Womans University, Seoul, Republic of Korea; 2 Milken Institute School of Public Health, George Washington University, Washington, DC USA; 3 School of Psychology and Public Health, La Trobe University, Melbourne, Australia; 4 Department of Public Health Science, Graduate School of Public Health, Seoul National University, Seoul, South Korea; 5 Institute of Health and Environment, Seoul National University, Seoul, South Korea

**Keywords:** Patent linkage, Linkage, Access to medicines, TRIPS-plus, Constructive ambiguity, Free trade agreements

## Abstract

**Background:**

The inclusion of patent linkage mechanisms in bilateral and plurilateral trade and investment agreements has emerged as a key element in the United States’ TRIPS-Plus intellectual property (IP) negotiating agenda. However, the provisions establishing patent linkage mechanisms in several agreements appear to reflect a degree of ambiguity, potentially enabling some flexibility in their implementation. In this study, we reviewed the features of the prototypic patent linkage mechanism established by the Hatch-Waxman Act in the United States, and compared these with the implementation of systems in three countries whose agreements with the US include patent linkage obligations. From these analyses, we draw lessons for moderating the impact of these mechanisms on access to generic medicines.

**Methods:**

We reviewed the features of the patent linkage mechanism in the US, and undertook a detailed analysis of relevant treaty provisions and the manner of implementation in Canada, Australia, and South Korea.

**Results:**

A key difference between the US implementation of patent linkage and that of its trading partners is the disparate treatment afforded to biologics. Because of the significant differences in the regulatory frameworks applying to small molecule and biologic medicines in the US, the Hatch- Waxman provisions do not apply to biologics and they are not subject to patent linkage. By contrast, the regulatory frameworks in Canada, Australia and South Korea do not reflect similar distinctions and thus patent linkage mechanisms also capture biologics. Additional variations in implementation, mainly the result of constructive ambiguities in the respective treaty texts, offer potential opportunity for mitigating the adverse impact of patent linkage provisions on market entry of generic medicines. Practical measures include ensuring the availability of an accessible, transparent and easily searchable database of patent information; avoiding automatic stays of generic marketing approval where possible; and requiring certification by rights holders to prevent abuse of the system.

**Conclusions:**

Where countries accept treaty obligations to establish patent linkage mechanisms, the impact on access to generic medicines may be moderated to a degree by retaining and exploiting constructive ambiguities in the treaty text and addressing practical aspects of implementation.

## Background

Patent linkage refers to the application of a conditional relationship between the granting of marketing approval for a generic medicine and the patent status of the originator reference product [1]. Patent linkage was first established in the United States (US) through the Drug Price Competition & Term Restoration Act of 1984, better known as the Hatch-Waxman Act. An applicant seeking marketing approval for a generic medicine must provide certification to the Food and Drug Administration (FDA) and notification to the market authorization holder that its marketing approval will not infringe any patents on the originator listed in the FDA ‘Orange Book’.^1^ The certification may prompt an action for infringement in response,^2^ which then triggers an automatic stay of generic marketing approval for up to 30 months (unless the litigation is concluded in the generic manufacturer’s favour within that time) [1–4].^3^ After 30 months the FDA is free to grant marketing approval of the generic but if the rights holder’s litigation subsequently succeeds the generic may be removed from the market and will be required to pay damages. Not surprisingly, patent linkage has been shown to have a detrimental effect on access to medicines [5–7], by delaying generic market entry and allowing the high prices of originator medicines to remain unrestrained by generic competition.

**Table 1:** The patent list, including submission of patent information

	United States		Canada	Australia	South Korea
	Small molecules	Biologics			
Including patents belonging to biologics	Separate system	Separate system	Yes	Yes	Yes
Patent list^a^	Yes	No	Yes	No	Yes
Patents that may be listed	1) drug substance 2) drug product (composition and formulation) 3) pharmaceutical use	–	1) medicinal ingredient 2) formulation 3) dosage form 4) pharmaceutical use	–	1) drug substance 2) composition 3) dosage form 4) pharmaceutical use
Timing of submission of patent information	With NDA or within 30 days of patent grant	–	With MA application or within 30 days of patent grant	–	Within 30 days of the date of MA or patent grant
Excluded patents	None if consistent with above list	–	Patents granted after MA	–	Patents granted after MA
Management of the list	FDA, with administrative process	–	MOH, with effective examination	_	MFDS, with effective examination
Amendments to the list	Possible, by the NDA applicant	–	Possible, by the NDA applicant or the Minister	_	Possible by the NDA applicant or MFDS
Deletion from the list	Possible, through counterclaim against patent litigation	–	Possible, by the Minister	–	Possible, by MFDS or the NDA applicant

^a^Specifically for the purpose of administering the patent linkage mechanism

**Table 2:** The notification process

	United States		Canada	Australia	South Korea
	Small molecules	Biologics			
The person who provides notice	An applicant arguing that the patent is invalid or will not be infringed (Paragraph IV)	An applicant submitting an application for licensure of a biological products as biosimilar or interchangeable	An applicant arguing that the patent is invalid or will not be infringed (An allegation)	An applicant with the 26B(1)(b) certificate, an applicant arguing that the patent will not be infringed is not required to give a notice to the patent holder	An applicant arguing that the patent is invalid or will not be infringed
Recipients of notification	Owner of the patent and the MA holder	The holder of biologic MA	The MA holder	The patent holder in cases where a patent will be infringed. The generic must certify to the TGA that the MAH has been notified	Owner of the patent and the MA holder
Timing of notification	Within 20 days after the date the is application filed	Within 20 days after the date the application is filed	–	After review of the application, but before marketing approval is granted	Within 20 days after the date the application is filed

**Table 3:** Stay of generic registration or sales

	United States		Canada	Australia	South Korea
	Small molecules	Biologics			
Requirements for stay in approval	Patent litigation	Patent litigation after notice of commercial marketing of the biological product	Make application to the Federal Court	–	Patent litigation and statement that the request is made with respect to a genuine duly registered patent
Timing of request	Within 45 days of the date of receipt	–	Within 45 days after the date of receipt	_	Within 45 days after the date of receipt
Judgment of request	–	Reviewed by the court	Reviewed by the court	_	Reviewed by the MFDS
Specific measures	Administrative process by the FDA, a 30-month automatic stay-in-approval	Judicial process, a preliminary injunction	Quasi-administrative process by the MOH, a 24-month stay-in-approval	Injunction may be sought against sale but no process to prevent grant of marketing approval	Administrative process by the MFDS, a 9-month stay of sales
The starting point of the measures	The date of receiving the notification	–	The date on which the action is brought	–	The date of receiving the notification
Penalty against a misused stay of marketing approval	–	–	Yes, an MAH must compensate for any damage to an ANDA applicant	Yes, patent holder must provide compensation for losses by generics company and the relevant jurisdiction if unsuccessful in litigation	–

**Table 4:** Exclusivity for the first generic market entrant

	United States		Canada	Australia	South Korea
	Small molecules	Biologics			
Requirements for exclusivity	be the first ANDA applicant arguing paragraph IV	N/A	N/A	–	(1) the first ANDA that has challenged a patent and obtained a favorable decision; or (2) the first ANDA that has filed a challenge within 14 days of the first challenge and has become the first to obtain a favorable decision (3) filed a challenge within 14 days of the first challenge and become the first to obtain a favourable decision
Specific measures	180-day exclusivity	N/A	N/A	N/A	9-month exclusivity, additional 2-month period is available to compensate for delayed marketing due to the reimbursement process
A starting point of the measures	the date of the first commercial marketing	N/A	N/A	N/A	the date of the marketing

Originator companies and their industry associations generally argue that patent- linkage is a rational means of ensuring that the regulatory agency does not promote infringement [8]. However, the absence of patent linkage does not prevent the intellectual property (IP) rights holder from taking infringement action or seeking injunctive relief, though in the US the rights holder is required to demonstrate the nature of the infringement against a potential infringer before a preliminary injunction will be granted. In other words, even where patent linkage does not exist, patent holders will generally be able to seek a preliminary injunction provided they can demonstrate the nature of the putative infringement. Despite this, the pharmaceutical industry argues that they cannot always obtain adequate measures against a potential infringer, preferring patent linkage as a safeguard that facilitates earlier and less costly resolution of patent disputes [9].

Providing for dispute resolution on patent infringement before the product in question is allowed to enter that market is an important tool. Postponing marketing approval for any generic product known by regulatory entities to be covered by a patent until expiration of the patent or the resolution of legitimate patent disputes (often referred to as linkage) is important. Such a mechanism provides a “procedural gate” or safeguard, because it ensures that drug regulatory entities do not inadvertently contribute to infringement of patent rights granted by another government entity by granting marketing rights to a competitor of the innovative company. Legal mechanisms that allow for early resolution of patent disputes before the generic product in question gains marketing approval avoid the need for complex litigation over damages for marketing an infringing product.

However, it is also argued that patent linkage mechanisms require regulatory authorities – traditionally only concerned with quality, efficacy and safety issues – to undertake functions for which they generally have no expertise. Moreover, patent linkage enables the rights holder to obtain a de facto injunction against a potential infringer without any evaluation of the merits of its claim [8] or the nature of the putative infringement. Empirical research has shown that patent linkage in the United States is highly effective in protecting originator products from competition and discouraging or delaying generic entry, and can extend the effective market monopoly well beyond the protection provided by the patent on the original product [10].

Above all, patent linkage changes the nature of patent law from a private right, where enforcement depends on the rights holder’s diligence, to a public right, where enforcement is undertaken by national authorities, financed by taxpayers. There is no provision describing patent linkage in the Trade-Related Aspects of Intellectual Property Rights (TRIPS) Agreement, which provides only for general enforcement of patent rights. Patent linkage is in fact very much a US construct, and is effectively precluded in the European Union by the EU medicines directive.^4^ As a form of IP protection, patent linkage introduces a potential and significant obstacle to the timely availability of generic medicines, but is less arguably less well understood than other TRIPS-Plus IP provisions. However, an understanding of the nature and risk of the mechanism is essential even for developing countries, as they may well face future pressure to introduce this and other TRIPS-Plus provisions as trade-offs for market access in future trade agreements.

To date, patent linkage provisions have appeared mainly in US trade agreements with high-income countries [4, 5, 11–14]. Canada, Australia, and South Korea (hereafter Korea) introduced forms of patent linkage in 1993, 2005, and 2015, respectively, as part of the implementation of their respective US FTAs [15, 16]. In addition, the Trans Pacific Partnership Agreement (TPP), originally negotiated by twelve countries including the US and several low and middle-income countries (LMICs), contained a patent linkage provision [4]. Although the United States withdrew from the TPP following the election of President Trump, the eleven remaining countries have retained the linkage provision in the revived TPP (now re-named the *Comprehensive and Progressive Agreement for Trans-Pacific Partnership*, or CPTPP), even while some other TRIPS-plus provisions have been suspended (New Zealand Ministry of Foreign Affairs and Trade). Given this, from a public health perspective there is growing concern about the impact of such provisions, particularly in LMICs [7, 17–21].

There are, however, some notable constructive ambiguities in the texts of the various bilateral and plurilateral trade and investment agreements [4]. Constructive ambiguities in international agreements were described by Henry Kissinger as ‘the deliberate use of ambiguous language in a sensitive issue to advance some political purpose’ [22, 23]. Ambiguity is used in negotiation as a means of concealing conflict, by leading parties to ascribe different meanings to the text at hand [24]. Patent linkage mechanisms may therefore evolve very differently where such ambiguities exist.

This study analyzes differences in the implementation of patent linkage mechanisms in selected countries, including the extent to which these are attributable to constructive ambiguities in trade agreement provisions. It draws lessons for the establishment of patent linkage in LMICs that may acquire future patent linkage obligations through bilateral or plurilateral trade and investment agreements, by identifying where textual ambiguities create flexibilities that minimize the capacity of patent linkage provisions to delay generic market entry.

## Method

As the origin of patent linkage, the US may be considered to have the prototypical mechanism. The other countries were selected as examples from which to observe differences, many of which arise by virtue of constructive ambiguities in trade agreement provisions [4]. For the comparative analysis the patent linkage mechanism was disaggregated into the following components: the patent list; the notification process; the stay of generic registration or sale; and exclusivity for the first generic entrant. Not all components exist in every country where a patent linkage mechanism has been introduced.

We undertook a thorough review of the literature, including published review articles, original articles, commentaries, and grey literature regarding patent linkage systems. Detailed analysis was then undertaken to identify differences in patent linkage regimes at two levels: reviewing the nature of the provisions of the relevant agreements, and the details of the associated domestic implementation. We searched for constructive ambiguities in the Australia US Free Trade Agreement (AUSFTA), Korea US Free Trade Agreement (KORUS), and the TPP/CPTPP texts and then analyzed differences in the practical implementation of patent linkage in the selected countries, drawing on relevant domestic legislation.^5^

## Results

### Patent linkage in the United States

The starting point for understanding the patent linkage system in the US is arguably the FDA *Orange Book*. When a manufacturer seeking marketing approval for a new chemical entity submits a New Drug Application (NDA) to the FDA, the application must also list the patents that the manufacturer wishes to claim on the drug.^6^ Once the FDA approves the drug, it lists the patents in the *Orange Book*. The *Orange Book* functions as an effective industry guide to drug patents, but not all patents may be listed. Patents that the FDA regards as covered by the statutory provisions include those that claim the active ingredient(s); drug product formulation/composition patents; use patents for particular approved indications or methods of use; and certain other patents as detailed on FDA Form 35,429.^7^ Patents for processes, packaging, metabolites, and intermediates may not be included.^7,^^8^ A drug’s *Orange Book* listing may evolve; the manufacturer may subsequently obtain additional patents after marketing approval has been granted and may submit them to the FDA for inclusion in the *Orange Book* within 30 days of patent grant. The FDA manages the list without active involvement^9^; amending or deleting information on the list is only possible by the patent applicant, or by an Abbreviated New Drug Application (generic) applicant through a counterclaim against infringement litigation. Importantly, the *Orange Book* provisions apply only to small molecule drugs; there is no equivalent to the *Orange Book* for biologics, even though in many other respects the obligations of patent linkage still apply.^10^

Within 20 days of the date of an Abbreviated New Drug Application (ANDA) filing, an applicant making a paragraph (iv) certification^11^ – that is, asserting that a relevant patent in the *Orange Book* is invalid or will not be infringed – is required to notify both the owner of the patent and the market authorization holder of the application.^12^ Similarly, an applicant submitting a biosimilar product for marketing approval is also required, within the same period of time, to notify the marketing authorization holder (MAH) of the biological reference product of its application.^13^

The stay of generic marketing approval for chemical entities is a legal obligation of the FDA. Within 45 days of the date of receipt of the notification the patent owner or the MAH notified of the ANDA may request the FDA to impose a stay of generic marketing approval. The FDA is then precluded from approving the ANDA for up to 30 months, unless non-infringement or invalidity is established earlier either by court judgment or patent expiry during the 30 months.^14^ The patent owner or MAH must pursue infringement litigation within the 45-day period, but merely bringing the patent suit, regardless of merit, guarantees the imposition of the stay. By contrast, the stay of approval for biosimilars is triggered without the involvement of the FDA; it is given effect by the granting of a preliminary injunction by the court.^15^

As an incentive to patent challenges, and to promote the timely availability of generics, the first to file a successful paragraph (iv) certification receives 180 days of marketing exclusivity –during which time the FDA may not approve another generic version of the same product [2, 25]. The 180-days starts from the earlier of the date of first commercial marketing *or* the date of the court decision finding that the patent is either invalid or not infringed by the generic.^16^

### Constructive ambiguities in trade agreement texts

The AUSFTA was signed in February 2004 and implemented the following year [26]. Article 17.10.4 (in the Intellectual Property chapter) of the AUSFTA provides for an attenuated or ‘weak’ form of patent linkage in two parts: a) measures in the marketing approval process to prevent a third party from marketing a product during the term of a patent without the consent of the patent owner; and b) a provision for the owner to be notified of a marketing approval request made during the term of a patent. This wording provided scope for Australia to implement a patent linkage mechanism in a very different way to the United States.

The KORUS FTA is bilateral trade agreement that was signed in 2007 but not ratified until 2012 [27]. Article 18.9.5 (in the Intellectual Property Rights chapter) of the KORUS FTA provides for a patent linkage system with three components: a) a list of patents to which patent linkage will be applied, b) a system of notification of the patent owner, and c) measures in marketing approval process to prevent other persons from gaining marketing approval. The mechanism prescribed in KORUS is much closer to that of the United States, although some differences remain, and are described in the next section of the paper.

Lastly, the TPP is a plurilateral trade agreement that was originally negotiated among 12 countries. Despite being a signatory to the agreement, President Trump withdrew the US in January 2017. Subsequently the remaining 11 countries decided to proceed despite the absence of the US, and concluded negotiations for the rebadged CPTPP in January 2018. While some of most controversial intellectual property provisions were suspended, patent linkage provisions remained in the agreed CPTPP text [28]. Article 18.53 (in the intellectual property chapter) of the CPTPP provides for two alternative approaches. The first includes a) a system to provide notice to a (patent or marketing authorization) holder or to allow a holder to be notified if a third party seeks to market a product during the term of an applicable patent and b) procedures (either judicial or administrative) and expeditious remedies for the timely resolution of disputes. The second option provides for an administrative (rather than judicial) mechanism that precludes the grant of marketing approval to a third party seeking to market a product subject to a patent, without the consent or acquiescence of the patent holder [28]. The final wording of the two options outlined in the TPP/CPTPP was clearly intended to accommodate, where possible, existing systems in the participating countries; considerable variation is allowed for, and only Brunei Darussalam, Malaysia and Vietnam are likely to require legislative amendments to implement these obligations [18].

### Variations among selected countries

As noted previously, a key difference observed in the Canadian, Australia, and Korean patent linkage mechanisms is absence of the distinction between small molecule and biologic medicines seen in the US. Other key variations that we observed may be mainly attributed to the ways in which ambiguities in the various agreements have been given effect, and pertain to the patent list arrangements, the notification processes, the imposition of a stay on the grant of marketing approval or in entry to market of generic medicines, and exclusivity for the first generic entrant.

### The patent list

The patent list is an important feature of most patent linkage systems, but is absent in (Table 1). In this respect the Australian patent linkage mechanism resembles that applying to biologics in the US. In theory, patent linkage may be invoked for any patent granted in relation to a medicine in Australia.^17^

The US (for small molecule medicines), Canada, and Korea all operate their lists differently. Variations are noted with respect to the patents that may be listed; the timing of submission of information; and the nature of the entity managing the list. In all of the selected countries (excluding Australia) patents covering 1) the drug substance, 2) the drug product including composition and formulation, and 3) the approved use may be listed and thereby ‘protected’ by the patent linkage mechanism.^18^ However, it is important to note that any patent, whether listed or not, may be the basis of an infringement dispute, irrespective of the existence or scope of a patent linkage mechanism.

The timing of the submission of information to the list also differs in Canada, Korea and the US. Information may only be submitted with the NDA in Canada^19^; whereas it may be submitted within 30 days of the date of the NDA approval in Korea.^20^ Unlike the US, patents granted after the date of submission of the NDA in Canada, or after the date of marketing approval in Korea, may not be listed. In addition, the lists are managed with effective audit and examination by the Ministry of Health in Canada^21^ and the Ministry of Food & Drug Safety (MFDS) in Korea.^22^ These regulatory authorities may delete irrelevant patents on the lists.

### The notification process

The processes for notifying marketing authorization and/or patent holders when an application for marketing approval is submitted for a generic or biosimilar drug do not differ significantly among the US, Canada, and (Table 2). In Australia, however, the notification system is very different. Under Section 26B of the Therapeutic Goods Act 1989, a sponsor seeking marketing approval of a generic or biosimilar medicine must certify either that (i) it will not market the drug in a manner that would infringe a valid patent *or* (ii) it has notified the rights holder of their intention to market the drug before the expiry of the patent term. This means that notifying the rights holder is not mandatory for those who argue that the generic application will not infringe a valid claim of the patent. Importantly, both certification and notification may be given after the application is reviewed by the TGA (but prior to the product being added to the Australian Register of Therapeutic Goods) as opposed to when the application is filed, which is common in the other countries. This means there is no effective delay between the timing of certification and the grant of marketing approval. In addition, the process is essentially passive in that the TGA does not make any adjudication of the veracity of the certification, and is required to ensure only that it has been received.

### Imposition of a stay on marketing approval or market entry of generic medicines

In relation to stays on marketing approval or market entry of generic medicines, the discussion addresses the following components: the overall process for obtaining a stay; specific measures for the provision of a stay; requirements for certification when seeking a stay; and penalty provisions for false or misleading (Table 3).

Compared with the mechanism in the US, the processes by which an originator may seek a stay in the approval of a generic application or on market entry of the generic are quite different in Canada, Australia, and Korea. First, the mechanism may be divided into judicial and administrative processes. In Australia, there is no administrative mechanism by which to seek or obtain a stay of marketing approval, and the only measures to prevent market entry are judicial determinations made without the involvement of the TGA^23^ – thus loosely resembling the system applying to biologics in the US. The TGA can proceed to register the generic or biosimilar regardless of whether the patent term has ended or not, and it is up to the patent holder to apply to the court for an injunction to prevent market entry or other allegedly infringing activities. By contrast, in Canada^24^ and Korea^25^ a stay of marketing approval may be sought via an administrative process with the involvement of the regulatory authorities. However, the court still plays a role in Canada, where the MAH must bring an action against the generic applicant in the Federal Court within 45 days of the date of receipt of a notification to obtain a declaration that the generic applicant infringed the patent. A direction precluding the granting of marketing approval for 24 months is made from the date on which the action is brought.^26^

Second, the specific measures vary considerably, with a 30-month stay-of-marketing approval for chemical entities in the United States, 24 months in Canada and 9 months in Korea; an indefinite stay on market entry triggered by preliminary injunctions in Australia (if granted by the court) and for biologics in the US, subject to the conclusion of judicial processes.^27^ The term of the stay ranges from 9 months in Korea to 30 months for small molecule drugs in the US. Furthermore, the measures differ substantially: a stay on marketing approval in the US, a stay on sales in Korea, but only a preliminary injunction on market entry, if granted by the court, in Australia, and in the US for biologics.

Third, some countries require reciprocal certification by the marketing authorization holder. In Australia, the MAH must certify to the TGA that the infringement proceedings are being commenced in good faith; that they have reasonable prospects of success; and that they will be conducted without unreasonable delay.^28^ In Korea, a statement that the request is made with respect to a genuine duly registered patent must also be provided.^29^

Last, there are penalty provisions applying to the making of a false or misleading request for a stay in Canada,^30^ and for false and misleading certification in Australia.^31^ In these countries, the MAH may be required to provide compensation for any damages caused by delays to generic market entry in these circumstances. In line with this, on the decision of the court, a MAH may be required to pay compensation to the Commonwealth, a State, or a Territory in Australia. These penalties were introduced as part of the AUSFTA implementing legislation in Australia, in an attempt to deter patent holders from pursuing vexatious litigation.

### Exclusivity for the first generic entrant

A period of exclusivity is provided for the first generic market entrant as part of the patent linkage mechanism in the US and Korea, although this is not mandated in any international trade (Table 4) [4]. In these countries, the patent linkage system works in two ways: it extends the exclusivity of originators by imposing a stay for generics and counters this by creating an incentive for generics to challenge weak patents [2].

In Korea, an applicant may be granted a 9-month period of exclusivity starting from the date of market entry of the generic, provided that (1) it is the first generic applicant that has challenged a patent and obtained a favorable decision or (2) it is the first generic applicant that filed a challenge within 14 days of the first challenge and has subsequently become the first to obtain a favorable decision.^32^ Compared with the system in the US, the requirements for exclusivity are more complex, and the term of exclusivity is longer.

## Discussion

Since the advent of the TRIPS Agreement, the US has sought to expand and extend intellectual property protections for medicines by pursuing TRIPS-Plus provisions such as patent linkage through the negotiation of bilateral and plurilateral trade and investment agreements. As a result, Australia and Korea reluctantly introduced patent linkage mechanisms in 2005 and 2015, respectively. Given the detrimental effect of patent linkage on access to medicines through delays in the marketing approval of generics enabling the maintenance of high prices of originator products, there has been growing concern about its potential impact in LMICs. However, constructive ambiguities in some trade agreement texts offer opportunities to attenuate the worst effects [4] particularly where lack of specificity exists, or alternative approaches are provided for in patent linkage provisions. Given this, we draw lessons from the experience of selected high-income countries for establishing minimally disruptive mechanisms in LMICs should they be obliged to do so under the provisions bilateral or plurilateral trade agreements to which they are parties. Of course, patent linkage mechanisms should be avoided where possible, as adequate IP protection can and (consistent with TRIPS) should be achievable through the private enforcement of IP rights through the judicial system. However, this study was undertaken to explore the opportunities created by constructive ambiguities in international agreements and highlight flexibilities available to countries that nevertheless find themselves obligated to establish patent linkage mechanisms.

A publicly accessible and easily searchable patent list or database is useful to enable generic applicants to obtain information about relevant patents, identify opportunities to challenge weak patents, and determine optimal timing for market entry. If such a list is not available, as in Australia, generic applicants must locate the relevant information through less readily accessible channels. Locating and interpreting this information, however, takes time, money, and specific expertise. Irrespective of whether a patent linkage mechanism is in place, national patents registries could be enhanced to identify clearly to which products particular patents are relevant. Rather than setting up a separate list specifically to support a patent linkage mechanism, a transparent and accessible national registry is to be preferred over separate lists managed by regulators. In line with this, a recent independent Pharmaceutical Patents Review in Australia recommended that the Australian Government establish such a transparent list [29], however, Medicines Australia, the peak industry body representing originator pharmaceutical companies, opposed its establishment arguing that any list should only be established in conjunction with a notification system similar to that in the US [30].

Where the patent list is specific to the patent linkage mechanism, patents included in the list should be verified and promptly deleted if the information is inaccurate or misleading. For instance, irrelevant patents may be deleted by the regulatory authority in Canada^33^and Korea.^34^ In addition, patents granted without a direct relation to marketing approval, for example those obtained post hoc, should not be permitted on the patent list; both Canada and Korea exclude patents granted after marketing authorization.

Market competitiveness is closely related to specific measures of patent linkage such as stay of generic registration. The patent linkage provisions of some trade agreements, including those in the TPP/CPTPP, do not require an automatic stay, thus allowing parties to implement the attenuated form of patent linkage found in Australia. In situations where an automatic stay must be provided, a stay applied to market entry rather than marketing approval would be recommended to mitigate further delays in the availability of the generic once the stay is complete or the IP issues resolved. In principle, the term of any stay should be as short as possible, and not granted automatically. The longer the stay, the greater the impact in delaying generic entry. In addition, certification is required from patent holders seeking a stay of approval or injunctive relief in some countries, and is recommended to discourage abuse of the process. False or misleading certification may also be linked to pecuniary penalties, as in Canada and Australia.^35^

It can be challenging, however, to introduce penalties of this type. In Korea, there have been several disputes over a provision requiring the rebate of profits acquired through a misleading or vexatious request for a stay of marketing approval for a generic [31]. Because an inappropriate stay would lead to financial losses to the health system, the National Health Insurance Service (NHIS) tried to introduce the penalty clause in an amendment to the National Health Insurance Act [32]. According to the draft, the NHIS could seek the return of the improper profit induced by the misleading request for the stay. The penalty system, which was to be established under the jurisdiction of the NHIS, differed from those in Canada and Australia which are under jurisdiction of the courts. The ambitious attempt to introduce penalties, however, failed. The National Assembly of the Republic of Korea (Assembly) thought that the penalty system might impede the capacity of patent holders to mount adequate defences against patent infringement.

The relationship between generic exclusivity and market competition should also be considered. The granting of exclusivity to the first generic entrant currently exists only in the United States and Korea, and in theory may be introduced with or without a patent linkage mechanism. Because of differences in pharmaceutical markets and policy frameworks, it is difficult to generalize the effects of such exclusivity. In principle, the entry of the first generic to the market will prompt rapid erosion in the originator’s market share, as occurs in the US [33], thus providing a strong incentive to for first mover status. Countries that want to encourage patent challenges against originators could consider administrative post-grant opposition procedures in lieu of full-blown judicial proceedings. Accelerating litigation, disallowing the presumption of validity, or fee shifting could be also considered. On the other hand, generic exclusivity may be less compelling in markets where price erosion is less pronounced (ie markets where the prices of originators and/or generics are closely regulated, or where therapeutic reference pricing is used) or where there is extensive marketing of authorized generics.^36^

The devil is, not surprisingly, in the detail. The appropriateness and manner of implementation of generic exclusivity should be determined in the context of the domestic pharmaceutical policy framework and local market conditions. For example, in Australia, where therapeutic reference pricing is used extensively, mandatory price reductions are applied to all versions of a drug on the formulary of the national drug coverage programme, the Pharmaceutical Benefits Scheme (PBS), when the first generic or biosimilar is added. [34]. Prices of all versions of the drug are then adjusted further as market competition prompts further price erosion [35]. Introducing generic exclusivity in a system such as this would be less effective as an inducement to challenge a patent.

Patent linkage mechanisms should be monitored, including keeping track of the number of requested stays (where relevant), the number of granted stays, and the actual terms of each stay. For example, Health Canada regularly releases the Therapeutic Products Directorate Statistical Report, which monitors the number of patents nominated, rejected, and added to the patent list; the number of ANDAs arguing patent invalidity; and the number of stay-in-approval requests [36]. Korea also amended the Pharmaceutical Affairs Act and introduced a mandatory impact analysis system under the supervision of the MFDS.^37^ Under the Act, the MFDS is required to monitor the operations of the system as is done in Canada, and evaluate the impacts of the patent linkage mechanism including the effect on pharmaceutical expenditure. The monitoring report and the impact analysis should be annually reported to the Assembly [37].

Lastly, any patent linkage mechanism should be harmonized with other national medicines policies. For example, there has been widespread concern that patent linkage might impede the use of compulsory licensing, despite being a recognised flexibility within TRIPS (and retained within CPTPP). While it would be convenient to assume that the right to grant a compulsory licence might also confer a right to override blocking patents, it is arguably more pragmatic to assume that a domestic court would find that an exception to patent linkage is not implied. To avert this risk, LMICs should ensure that essential exemptions to patent linkage are provided for in domestic legislation.

## Conclusions

Since TRIPS the US has negotiated a series of bilateral and plurilateral trade agreements that have emphasised ‘TRIPS-Plus’ IP provisions, and in some cases have included requirements for trading partners to establish patent linkage mechanisms. However, there are notable constructive ambiguities in some agreement texts, offering avenues for flexibility and discretion in implementing certain features of the system domestically.

Several key features of the patent linkage mechanisms implemented in Canada, Australia and Korea deviate significantly from the US system, and many of these deviations are likely to mitigate the effects of patent linkage on the timing of generic entry and on access to medicines. These include: variations in the patent list (whether there is a list at all and if so, how it is managed), the type of notification system and timing of notification; whether the stay is on marketing approval or sales, and the length of the stay; and whether exclusivity for the first generic entrant is provided. Variations in the implementation of patent linkage in Australia and Korea arise from constructive ambiguities in the AUSFTA and KORUS. The patent linkage provisions of the CPTPP also provide considerable scope for parties to retain their existing mechanisms (for those countries such as Australia and Canada which already have patent linkage) or to implement mechanisms which are different to the US model in important ways.

This is the first study to examine the different approaches in the US, Canada, Australia, and Korea, and identify insights relevant to LMICs that may be forced to introduce the mechanism in the future, To date there have been no studies comprehensively describing the heterogeneity of the mechanisms across different countries. We have endeavoured to identify both obvious and more nuanced differences in implementation in countries that have agreed to implement this TRIPS-Plus measure. Drawing lessons from the experiences of countries with these mechanisms can be salutary, and useful in informing approaches that could mitigate to a degree the detrimental effects of patent linkage – delayed access to generic medicines and sustained high prices of patented products.

## Abbreviations

ANDA: Abbreviated New Drug Application
Assembly: National Assembly of the Republic of Korea
AUSFTA: Australia-United States Free Trade Agreement
CPTPP: Comprehensive and Progressive Agreement for Trans-Pacific Partnership
FDA: Food and Drug Administration
KORUS FTA: Korea-United States Free Trade Agreement
LMIC: Low- and middle-income countries
MAH: Marketing Authorization Holder
MFDS: Ministry of Food and Drug Safety
NDA: New Drug Application
NHIS: National Health Insurance Service
TGA: Therapeutic Goods Administration
TPPA: Trans Pacific Partnership Agreements
TRIPS: Agreement on Trade-related Aspects of Intellectual Property Rights

## Footnotes

1 The *Approved Drug Products with Therapeutic Equivalence Evaluations*

2 If the litigation is not initiated within 45 days the FDA is free to grant marketing approval, but the generic launch may nevertheless be ‘at risk’.

3 Notably, prior to the Medicare Prescription Drug Improvement and Modernization Act of 2003 multiple 30-month stays were possible.

4 The processing of marketing authorization procedures can be carried out without being affected by the protection of industrial and commercial property interests. See Directive 2001/83/EC of the European Parliament and of the Council of 6 November 2001 on the *Community Code Relating to Medicinal Products for Human Use*

5 The Drug Price Competition & Patent Term Restoration Act 1984, the Medicare Prescription Drug, Improvement, & Modernization Act 2003, and the Biologics Price Competition & Innovation Act 2009 in the US, the Patented Medicines Notice of Compliance (PMNOC) Regulation in Canada, the Therapeutic Goods Act 1989 in Australia, and the Pharmaceutical Affairs Act in Korea.

6 Code of Federal Regulations Title 21, Sec 314.53 (d) When and where to submit patent information

7 Code of Federal Regulations Title 21, Sec 314.53 (b) Patents for which information must be submitted and patents for which information must not be submitted

8 Code of Federal Regulations Title 21, Sec 314.53 (c) Reporting requirements

9 Code of Federal Regulations Title 21, Sec 314.53 (f) Correction of patent information error

10 42 U.S.C. § 262 (l) Patents

11 21 U.S.C. § 355 (b) Filing Application. An ANDA submission requires the filing of a certificate asserting that either

- para (i) - no patent is listed in the Orange Book
- para (ii) – the listed patent has expired
- para (iii) – the listed patent will expire before approval is granted
- para (iv) – the listed patent is invalid or will not be infringed.

Even though the generic manufacturer cannot market the drug prior to approval of the ANDA, the filing of a Paragraph IV Certification is nonetheless treated as an act of patent infringement (35 U.S.C. § 271(e)(2)(A)

12 21 U.S.C. § 355 (j) Abbreviated New Drug Applications

13 42 U.S.C. § 262 (l) Patents

14 21 U.S.C. § 355 (j) Abbreviated New Drug Applications

15 42 U.S.C. § 262 (l) Patents

16 21 U.S.C. § 355 (j) Abbreviated New Drug Applications

17 Therapeutic Goods Act 1989, s.26B

18 Code of Federal Regulations Title 21, Sec 314.53 (c) Reporting requirements in the United States, PMNOC 4 (2) in Canada, the Pharmaceutical Affairs Act 50–2 in Korea

19 PMNOC 4

20 The Pharmaceutical Affairs Act 50–3

21 PMNOC 3.1

22 The Pharmaceutical Affairs Act 50–3

23 Therapeutic Goods Act 1989 26C and 26D

24 PMNOC 7 (1)

25 The Pharmaceutical Affairs Act 50–6

26 PMNOC 7 (1)

27 In this context the Australian system does not in fact meet the definition of patent linkage since there is no direct link between the patent status of the originator and the marketing approval of the generic.

28 Therapeutic Goods Act 26C (3)

29 The Pharmaceutical Affairs Act 50–5

30 PMNOC 8

31 Therapeutic Goods Act 26C and 26D

32 The Pharmaceutical Affairs Act 50–7

33 PMNOC 3

34 The Pharmaceutical Affairs Act 50–3

35 Australia imposes pecuniary penalties for false or misleading certification, but not for failure to certify.

36 An authorized generic (or pseudo-generic) is an originator’s prescription drug that is marketed and distributed by the originator or under license by an authorized generic distributor with a generic product label.

37 The Pharmaceutical Affairs Act 50–11
